# Supplementary material for: Meiofauna at a tropical sandy beach in the SW Atlantic: the influence of seasonality on diversity
Source: PeerJ. 2024 Jul 12;12:e17727. doi: 10.7717/peerj.17727 (PMC11249015; doi:10.7717/peerj.17727)
Supplement: Supplemental Information 1 [file peerj-12-17727-s001.docx]

| Season | Date |
| --- | --- |
| Summer | December 12^th^, 2019  January 12^th^, 2020  February 11^th^, 2020 |
| Autumn | March 11^th^, 2020  April 7^th^, 2020  May 26^th^, 2020 |
| Winter | June 22^nd^, 2020  July 23^rd^, 2020  August 22^nd^, 2020 |
| Spring | September 20^th^, 2020  October 15^th^, 2020  November 28^th^, 2020 |
