# Supplementary material for: Meiofauna at a tropical sandy beach in the SW Atlantic: the influence of seasonality on diversity
Source: PeerJ. 2024 Jul 12;12:e17727. doi: 10.7717/peerj.17727 (PMC11249015; doi:10.7717/peerj.17727)
Supplement: Supplemental Information 2 [file peerj-12-17727-s002.docx]

| Sample ID | Input | Filtered | Input passed filter (%) | Denoised | Non-chimeric | Input non-chimeric (%) |
| --- | --- | --- | --- | --- | --- | --- |
| Summer1 | 57335 | 4717 | 8.23 | 4435 | 4267 | 7.44 |
| Summer2 | 67691 | 7653 | 11.31 | 7290 | 7110 | 10.5 |
| Summer3 | 69365 | 5787 | 8.34 | 5473 | 5299 | 7.64 |
| Summer4 | 76136 | 6670 | 8.76 | 6224 | 6049 | 7.94 |
| Summer5 | 65959 | 5772 | 8.75 | 5315 | 5218 | 7.91 |
| Summer6 | 68503 | 6556 | 9.57 | 5989 | 5835 | 8.52 |
| Summer7 | 69444 | 5916 | 8.52 | 5528 | 5359 | 7.72 |
| Summer8 | 75647 | 9623 | 12.72 | 9126 | 8765 | 11.59 |
| Summer9 | 73658 | 6310 | 8.57 | 5929 | 5790 | 7.86 |
| Autumn1 | 61173 | 5764 | 9.42 | 5382 | 5315 | 8.69 |
| Autumn2 | 72346 | 9205 | 12.72 | 8763 | 8591 | 11.87 |
| Autumn3 | 68942 | 6589 | 9.56 | 6150 | 5982 | 8.68 |
| Autumn4 | 54009 | 4633 | 8.58 | 3983 | 3926 | 7.27 |
| Autumn5 | 56085 | 5724 | 10.21 | 5036 | 5036 | 8.98 |
| Autumn6 | 67106 | 5858 | 8.73 | 5198 | 5038 | 7.51 |
| Autumn7 | 61996 | 5598 | 9.03 | 4967 | 4853 | 7.83 |
| Autumn8 | 69247 | 7048 | 10.18 | 6521 | 6304 | 9.1 |
| Autumn9 | 66745 | 5770 | 8.64 | 5142 | 5018 | 7.52 |
| Winter1 | 135592 | 15161 | 11.18 | 14419 | 13399 | 9.88 |
| Winter2 | 66014 | 6248 | 9.46 | 5760 | 5696 | 8.63 |
| Winter3 | 64426 | 6135 | 9.52 | 5392 | 5233 | 8.12 |
| Winter4 | 66217 | 11588 | 17.5 | 10965 | 10781 | 16.28 |
| Winter5 | 67431 | 6628 | 9.83 | 5811 | 5706 | 8.46 |
| Winter6 | 64384 | 7090 | 11.01 | 6452 | 6367 | 9.89 |
| Winter7 | 57765 | 5910 | 10.23 | 5315 | 5197 | 9.00 |
| Winter8 | 59813 | 7441 | 12.44 | 6831 | 6795 | 11.36 |
| Winter9 | 54150 | 8739 | 16.14 | 7953 | 7902 | 14.59 |
| Spring1 | 71363 | 38458 | 53.89 | 37764 | 37130 | 52.03 |
| Spring2 | 76540 | 54401 | 71.08 | 53353 | 52528 | 68.63 |
| Spring3 | 2115 | 1842 | 87.09 | 1384 | 1384 | 65.44 |
| Spring4 | 67197 | 35857 | 53.36 | 35129 | 35030 | 52.13 |
| Spring5 | 70811 | 20802 | 29.38 | 20164 | 19511 | 27.55 |
| Spring6 | 70582 | 40547 | 57.45 | 39925 | 39767 | 56.34 |
| Spring7 | 65500 | 54743 | 83.58 | 54167 | 54167 | 82.7 |
| Spring8 | 65178 | 51271 | 78.66 | 50637 | 49270 | 75.59 |
| Spring9 | 67830 | 57126 | 84.22 | 56553 | 54429 | 80.24 |
