# Supplementary material for: Meiofauna at a tropical sandy beach in the SW Atlantic: the influence of seasonality on diversity
Source: PeerJ. 2024 Jul 12;12:e17727. doi: 10.7717/peerj.17727 (PMC11249015; doi:10.7717/peerj.17727)
Supplement: Supplemental Information 3 — Permutational Multivariate Analysis of Variance results from environmental data (rainfall. grain size, carbonate, organic matter, biopolymers) collected in Gramuté beach, SE Brazil, during all seasons (summer, autumn, winter, and spring). Significative results are considered when p<0.05, and are presented in bold. df = Degrees of Freedom; SS = Sum of Squares; MS = Mean of Squares. [file peerj-12-17727-s003.docx]

| Source | df | | SS | MS | Pseudo-F | | *p* |
| --- | --- | --- | --- | --- | --- | --- | --- |
| Season | 3 | | 123.9 | 41.3 | 6.916 | | **0.001** |
| Residual | 32 | | 191.1 | 5.97 |  | |  |
| Total | 35 | | 315.0 |  |  | |  |
| Pair-wise tests | | | | | | | |
| Groups | | t | | | | *p* | |
| Summer X Autumn | | 2.698 | | | | **0.001** | |
| Summer X Winter | | 2.874 | | | | **0.001** | |
| Summer X Spring | | 2.632 | | | | **0.005** | |
| Autumn X Winter | | 1.923 | | | | **0.027** | |
| Autumn X Spring | | 3.009 | | | | **0.001** | |
| Winter X Spring | | 2.476 | | | | **0.002** | |
