# Supplementary material for: Meiofauna at a tropical sandy beach in the SW Atlantic: the influence of seasonality on diversity
Source: PeerJ. 2024 Jul 12;12:e17727. doi: 10.7717/peerj.17727 (PMC11249015; doi:10.7717/peerj.17727)
Supplement: Supplemental Information 4 — Relative abundance of meiofaunal taxa (until Order) from sediment samples collected at Gramuté beach, SE Brazil, during all seasons (summer, autumn, winter, and spring). [file peerj-12-17727-s004.docx]

| Phyllum | Class | Order | Season | | | |
| --- | --- | --- | --- | --- | --- | --- |
|  |  |  | Summer | Autumn | Winter | Spring |
| Annelida miscellaneous |  |  | 0.632% | 0.688% | 0.285% | 1.868% |
|  | Clitellata | Haplotaxida | 0.153% | 0.000% | 0.018% | 0.000% |
|  | Polychaeta miscellaneous |  | 0.250% | 0.000% | 0.137% | 0.177% |
|  |  | Aspidosiphonidormes | 0.000% | 0.021% | 0.000% | 0.000% |
|  |  | Capitellida | 2.979% | 2.472% | 0.186% | 0.000% |
|  |  | Echiuroinea | 0.233% | 0.348% | 0.120% | 0.039% |
|  |  | Eunicida | 0.113% | 0.225% | 0.345% | 0.000% |
|  |  | Golfingiida | 0.000% | 0.000% | 0.042% | 0.000% |
|  |  | Phyllodocida | 0.097% | 0.017% | 0.077% | 0.334% |
|  |  | Protodrilidae | 0.000% | 0.000% | 0.000% | 0.098% |
|  |  | Sabellida | 0.000% | 0.025% | 0.011% | 0.000% |
|  |  | Spionida | 0.000% | 0.000% | 0.000% | 0.079% |
|  |  | Terebellida | 0.000% | 0.000% | 0.011% | 0.393% |
| Arthropoda (Crustacea) | Malacostraca | Eucarida | 0.000% | 0.008% | 0.000% | 0.079% |
|  | Maxillopoda miscellaneous |  | 1.292% | 0.268% | 2.057% | 0.511% |
|  |  | Calanoida | 0.000% | 0.000% | 0.011% | 0.000% |
|  |  | Harpacticoida | 2.433% | 0.888% | 3.646% | 0.865% |
|  | Ostracoda | Podocopida | 0.186% | 3.631% | 0.580% | 4.444% |
| Cnidaria | Anthozoa | Actinaria | 0.117% | 0.000% | 0.025% | 0.000% |
|  |  | Zoantharia | 0.000% | 0.000% | 0.000% | 0.079% |
| Echinodermata | Echinoidea |  | 0.017% | 0.000% | 0.063% | 0.000% |
|  | Holothuroidea |  | 0.037% | 0.340% | 0.000% | 0.079% |
|  | Ophiuroidea |  | 0.000% | 0.051% | 0.000% | 0.000% |
| Gastrotricha |  | Chaetonotida | 0.000% | 0.000% | 0.141% | 0.000% |
|  |  | Macrodasyida | 0.206% | 0.021% | 0.239% | 0.000% |
| Mollusca | Bivalvia | Mytiloida | 0.013% | 0.021% | 0.032% | 0.000% |
|  |  | Ostreoida | 0.000% | 0.017% | 0.000% | 0.000% |
|  |  | Pterioida | 0.007% | 0.055% | 0.084% | 0.216% |
|  | Gastropoda | Caenogastropoda | 0.107% | 0.055% | 0.000% | 0.000% |
|  |  | Heterobranchia | 0.010% | 0.000% | 0.000% | 0.000% |
|  |  | Vetigastropoda | 0.013% | 0.013% | 0.000% | 0.000% |
| Nematoda | Chromadorea miscellaneous |  | 1.039% | 0.437% | 1.220% | 0.157% |
|  |  | Chromadorida | 0.030% | 0.017% | 0.000% | 0.000% |
|  |  | Desmodorida | 0.017% | 0.000% | 0.000% | 0.000% |
|  |  | Monhysterida | 0.186% | 0.000% | 0.000% | 0.000% |
|  |  | Rhabditida | 0.273% | 0.013% | 0.721% | 0.000% |
|  | Enoplea | Enoplida | 0.000% | 0.038% | 0.000% | 0.000% |
| Nemertea | Anopla | Paleonemertea | 0.000% | 0.000% | 0.000% | 0.393% |
|  | Enopla | Monostilifera | 0.030% | 0.000% | 0.120% | 0.000% |
| Platyhelminthes miscellaneous |  |  | 0.000% | 0.059% | 0.000% | 0.000% |
|  | Rhabditophora | Rhabdocoela | 0.632% | 1.346% | 0.942% | 0.629% |
| Rotifera | Bdelloidea |  | 0.000% | 0.034% | 0.000% | 0.000% |
