# Supplementary material for: Meiofauna at a tropical sandy beach in the SW Atlantic: the influence of seasonality on diversity
Source: PeerJ. 2024 Jul 12;12:e17727. doi: 10.7717/peerj.17727 (PMC11249015; doi:10.7717/peerj.17727)
Supplement: Supplemental Information 5 — Results from Similarity Percentage analysis (SIMPER) indicating each taxon contribution to the dissimilarity among seasons (pair-wise comparisons) in Gramuté beach, SE Brazil. Av. Dissim. = Average Dissimilarity; Diss./SD = Dissimilarity/Standard Deviation; Contrib. = Contribution. [file peerj-12-17727-s005.docx]

| Seasonal Comparison | | | | |
| --- | --- | --- | --- | --- |
| Taxon |  | Autumn vs Spring (Av. Diss. = 49.45%) | | |
|  | Av. Diss. | Diss./SD | Contribution (%) | Cum.Contrib. (%) |
| Crustacea | 13.19 | 1.56 | 26.66 | 26.66 |
| Annelida | 11.12 | 1.08 | 22.49 | 49.15 |
| Platyhelminthes | 7.61 | 1.09 | 15.39 | 64.54 |
| Nematoda | 7.53 | 1.40 | 15.24 | 79.78 |
| Gastrotricha | 2.79 | 1.01 | 5.65 | 85.43 |
| Mollusca | 2.79 | 1.19 | 5.63 | 91.06 |
| Echinodermata | 2.55 | 0.82 | 5.16 | 96.23 |
| Cnidaria | 0.84 | 0.35 | 1.69 | 97.92 |
| Rotifera | 0.59 | 0.34 | 1.18 | 99.11 |
| Nemertea | 0.44 | 0.35 | 0.89 | 100.00 |
| Autumn vs Summer (Av.Diss. = 49.71%) | | | | |
|  | Av. Diss. | Diss./SD | Contribution (%) | Cum.Contrib. (%) |
| Annelida | 11.28 | 1.13 | 22.70 | 22.70 |
| Crustacea | 10.82 | 1.28 | 21.76 | 44.46 |
| Nematoda | 7.59 | 1.09 | 15.27 | 59.73 |
| Platyhelminthes | 7.01 | 1.09 | 14.09 | 73.82 |
| Gastrotricha | 3.69 | 1.16 | 7.42 | 81.24 |
| Cnidaria | 2.56 | 0.61 | 5.14 | 86.38 |
| Mollusca | 2.44 | 1.19 | 4.90 | 91.28 |
| Nemertea | 2.09 | 0.71 | 4.21 | 95.49 |
| Echinodermata | 1.27 | 0.75 | 2.55 | 98.04 |
| Rotifera | 0.97 | 0.35 | 1.96 | 100.00 |
| Spring vs Summer (Av.Diss. = 56.00%) | | | | |
|  | Av. Diss. | Diss./SD | Contribution (%) | Cum.Contrib. (%) |
| Crustacea | 14.01 | 1.29 | 25.02 | 25.02 |
| Annelida | 11.39 | 1.26 | 20.35 | 45.36 |
| Nematoda | 8.99 | 0.84 | 14.88 | 60.24 |
| Platyhelminthes | 7.48 | 0.66 | 13.36 | 73.60 |
| Gastrotricha | 3.67 | 0.87 | 6.55 | 80.15 |
| Mollusca | 2.56 | 1.15 | 4.56 | 84.71 |
| Nemertea | 2.37 | 0.65 | 4.24 | 88.95 |
| Echinodermata | 2.31 | 0.67 | 4.12 | 93.07 |
| Cnidaria | 2.26 | 0.50 | 4.04 | 97.10 |
| Rotifera | 1.62 | 0.49 | 2.90 | 100.00 |
| Autumn vs Winter (Av.Diss. = 66.70%) | | | | |
|  | Av. Diss. | Diss./SD | Contribution (%) | Cum.Contrib. (%) |
| Annelida | 17.12 | 0.91 | 25.67 | 25.67 |
| Crustacea | 16.77 | 1.69 | 25.14 | 50.81 |
| Nematoda | 12.93 | 1.76 | 19.38 | 70.19 |
| Platyhelminthes | 7.84 | 1.34 | 11.75 | 81.94 |
| Mollusca | 3.55 | 1.13 | 5.32 | 87.26 |
| Gastrotricha | 3.50 | 1.00 | 5.24 | 92.51 |
| Nemertea | 1.79 | 0.60 | 2.68 | 95.19 |
| Echinodermata | 1.66 | 0.72 | 2.49 | 97.68 |
| Cnidaria | 1.55 | 0.54 | 2.32 | 100.00 |
| Spring vs Winter (Av.Diss = 62.91%) | | | | |
|  | Av. Diss. | Diss./SD | Contribution (%) | Cum.Contrib. (%) |
| Annelida | 17.78 | 0.97 | 28.26 | 28.26 |
| Crustacea | 16.36 | 1.14 | 26.01 | 54.27 |
| Nematoda | 8.75 | 1.28 | 13.91 | 68.18 |
| Platyhelminthes | 8.35 | 0.65 | 13.28 | 81.45 |
| Mollusca | 4.14 | 1.08 | 6.57 | 88.03 |
| Echinodermata | 3.22 | 0.70 | 5.12 | 93.15 |
| Nemertea | 1.84 | 0.50 | 2.93 | 96.07 |
| Rotifera | 1.02 | 0.34 | 1.62 | 97.69 |
| Cnidaria | 0.78 | 0.50 | 1.25 | 98.94 |
| Gastrotricha | 0.67 | 0.34 | 1.06 | 100.00 |
| Summer vs Winter (Av.Diss = 68.62%) | | | | |
|  | Av. Diss. | Diss./SD | Contribution (%) | Cum.Contrib. (%) |
| Crustacea | 18.32 | 1.24 | 26.69 | 26.69 |
| Nematoda | 14.98 | 0.90 | 21.83 | 48.52 |
| Annelida | 11.35 | 1.14 | 16.54 | 65.06 |
| Platyhelminthes | 6.71 | 0.55 | 9.78 | 74.84 |
| Gastrotricha | 4.75 | 0.85 | 6.92 | 81.76 |
| Nemertea | 3.80 | 0.78 | 5.54 | 87.31 |
| Mollusca | 3.34 | 1.02 | 4.86 | 92.17 |
| Cnidaria | 3.09 | 0.59 | 4.50 | 96.67 |
| Rotifera | 1.29 | 0.35 | 1.89 | 98.55 |
| Echinodermata | 0.99 | 0.49 | 1.45 | 100.00 |
